# Musicianship Influences Language Effect on Musical Pitch Perception

**DOI:** 10.3389/fpsyg.2021.712753

**Published:** 2021-10-04

**Authors:** William Choi

**Affiliations:** Academic Unit of Human Communication, Development, and Information Sciences, The University of Hong Kong, Hong Kong, SAR China

**Keywords:** OPERA, pitch, tone, rhythm, language-to-music transfer

## Abstract

Given its practical implications, the effect of musicianship on language learning has been vastly researched. Interestingly, growing evidence also suggests that language experience can facilitate music perception. However, the precise nature of this facilitation is not fully understood. To address this research gap, I investigated the interactive effect of language and musicianship on musical pitch and rhythmic perception. Cantonese and English listeners, each divided into musician and non-musician groups, completed the Musical Ear Test and the Raven’s 2 Progressive Matrices. Essentially, an interactive effect of language and musicianship was found on musical pitch but not rhythmic perception. Consistent with previous studies, Cantonese language experience appeared to facilitate musical pitch perception. However, this facilitatory effect was only present among the non-musicians. Among the musicians, Cantonese language experience did not offer any perceptual advantage. The above findings reflect that musicianship influences the effect of language on musical pitch perception. Together with the previous findings, the new findings offer two theoretical implications for the OPERA hypothesis—bi-directionality and mechanisms through which language experience and musicianship interact in different domains.

## Introduction

Long-term musical experience facilitates speech perception (Pfordresher and Brown, 2009; Bidelman et al., 2010). This effect, known as *music-to-language transfer*,^1^ largely undergirds theoretical models of cross-domain plasticity (Patel, 2011, 2012, 2014; Krishnan et al., 2012). Interestingly, there is emerging evidence of *language-to-music transfer* (Bidelman et al., 2013; Chen et al., 2016; Zhang et al., 2020). These studies generally showed that tone language experience enhanced musical pitch perception among non-musicians. However, these novel findings could not situate well in the OPERA hypothesis as it was designed for music-to-language transfer. Also, the OPERA hypothesis does not embody the interaction between musicianship and language experience, presumably because very few studies systematically manipulated both variables together (Cooper and Wang, 2012; Ngo et al., 2016; Maggu et al., 2018b). Apart from pitch, rhythm is also a common acoustic feature of music and speech (Zhang et al., 2020). As such, Patel (2012) called for future studies to extend the OPERA hypothesis from pitch to rhythm. To broaden the OPERA hypothesis, the current study examined the interactive effects of musicianship and language experience on musical pitch and rhythmic perception.

The OPERA hypothesis theorizes how long-term musical experience increases neuronal sensitivity to perceptual attributes in the language domain, most notably tones (Patel, 2011). In the hypothesis, a clear conceptual distinction was made between perceptual attributes (e.g., tones and musical pitch) and acoustic features (e.g., periodicity). According to Patel (2011), music-to-language transfer will occur only when five conditions are met—Overlap, Precision, Emotion, Repetition, and Attention. Regarding Overlap, although different perceptual attributes (tones and musical pitch) are processed differently at the cortical level, the processing of their acoustic feature (i.e., periodicity) recruit overlapping subcortical networks. For Precision, music must require more nuanced processing than speech. For Emotion, strong positive emotion must be brought about by musical activities. In terms of Repetition, there must be a frequent repetition of the musical activities. For Attention, the musical activities must require focused attention. When the above conditions are met, musical experience will enhance neuronal precision in the subcortical area shared by music and language. Enhanced subcortical processing of the acoustic feature (i.e., periodicity) will in turn facilitate the processing of the linguistic perceptual attribute (i.e., tones).

### Music-to-Language Transfer

There is mounting cross-sectional evidence that musicianship facilitates tone perception in different tasks, e.g., discrimination, identification, sequence recall, and word learning. Concerning discrimination, English musicians discriminated Mandarin tones more accurately than did English non-musicians (Alexander et al., 2005). On the one hand, this result implied that musicianship facilitated English listeners’ tone discrimination. On the other hand, the perceptual facilitation might be speech general rather than specific to tones. In a later study, Italian musicians, Italian non-musicians, and Italian learners of Mandarin were presented with monosyllabic Mandarin word sequences with tonal and segmental violations (Delogu et al., 2010). Compared with the non-musicians, the musicians only detected tonal variations more accurately. This suggested that the music-to-language transfer was specific to tones. In a more recent study, English listeners heard pairs of Mandarin phrases, half of which contained a syllable with a deviant tone (i.e., the f0 level of the syllable was increased by 10%) (Zheng and Samuel, 2018). Compared with the English non-musicians, the English musicians were better able to detect the tonal differences. This indicated that music-to-language transfer was not limited to isolated words. As the above studies only used Mandarin tones, it remained unclear whether music-to-language transfer applied to more complex tone systems such as Cantonese (for a review of Cantonese tonal complexity, see Yip, 2002; Gu et al., 2007). In a Cantonese tone discrimination task, English musicians outperformed English non-musicians in half of the possible Cantonese tonal contexts (Choi, 2020). Despite the subtle differences between the tone discrimination studies, they generally provided evidence of music-to-language transfer. Remarkably, this transfer was not limited to Mandarin.

Music-to-language transfer also applied to tone identification and sequence recall. Following a brief familiarization of Mandarin tones, English musicians identified the Mandarin tones more accurately than did English non-musicians (Alexander et al., 2005; Lee et al., 2014). Critically, some tone identification studies reported the lack of correlation between musical pitch identification (i.e., absolute pitch) and Mandarin tone identification tasks (Lee and Lee, 2010; Lee et al., 2011, 2014). Does this lack of correlation indicate the absence of music-and-language relationship? In the only study which included English musicians and non-musicians, the musicians showed superior performance on Mandarin tone identification (Lee et al., 2014). Thus, the lack of correlation should not be taken to indicate the absence of music-to-language transfer. Instead, it merely reflected that the music-to-language transfer was not because the musicians had employed the perceptual mechanism of absolute pitch for Mandarin tone identification. In particular, enhanced neural encoding of periodicity might underlie a perceptual advantage on Mandarin tones (Patel, 2011, 2014). Going beyond identification, a recent study compared English musicians and non-musicians on their ability to recall Cantonese tone sequences (Choi, 2020). The English musicians outperformed the non-musicians on recalling contour tone sequences, indicating the presence of music-to-language transfer at the higher perceptual levels. Here, higher perceptual levels refer to the relative levels at which the perceptual operations are more complex than basic perceptual operations (e.g., forming phonological representations vs. judging the loudness of two beeps).

Concerning the higher perceptual levels, music-to-language transfer was also evident in tone-word learning. In a Mandarin tone-word learning experiment, English musicians and non-musicians were classified as successful (95% accuracy or above for two consecutive sessions) or less successful (less than 5% improvement for four consecutive sessions) learners (Wong and Perrachione, 2007). While only 22% of the non-musicians reached the successful criterion, as many as 88% of the musicians were classified as successful learners. Despite its small sample size (*n* = 17), the study provided initial evidence that music-to-language transfer applied to tone-word learning. With a more adequate sample size (*n* = 54), a later study compared English musicians, English non-musicians, Thai musicians, and Thai non-musicians on Cantonese tone word learning (Cooper and Wang, 2012). After training, the English musicians identified the tone words more accurately than did the English non-musicians. This convincingly reflected that music-to-language transfer was potent at the linguistic level, i.e., formation and recall of phonological-semantic links.

Aside behavioral evidence, there is ample neural evidence of music-to-language transfer (e.g., Wong et al., 2007; Bidelman et al., 2010; cf. Maggu et al., 2018a). At the subcortical level, English musicians showed stronger fundamental frequency-following response (FFR) to Mandarin tonal changes than English non-musicians (Wong et al., 2007). In a later study, English musicians even encoded two sections of the Mandarin rising tone more robustly than did Mandarin listeners (Bidelman et al., 2010). The above findings situated well in the OPERA hypothesis—musical experience strengthens the subcortical neural network shared by music and language; and the enhancement of the subcortical plasticity was leveraged for tone perception (Patel, 2011, 2014).

### Language-to-Music Transfer

Originally devised to account for music-to-language transfer, the OPERA hypothesis did not explicitly articulate about bidirectionality (Patel, 2011, 2012, 2014; see Asaridou and McQueen, 2013). Recall the Precision condition—for language-to-music transfer to occur, language must entail more precise pitch processing than music. However, Patel (2014) has argued that music requires finer pitch distinctions than language does—one semitone difference is perceptually salient in musical notes but not in lexical tones (Peretz and Hyde, 2003; Zatorre and Baum, 2012). Pertaining to the Emotion condition, Asaridou and McQueen (2013) believed that emotional reinforcement of speaking a tone language was hardly comparable to that of musical activities. As such, the authors reasoned that the OPERA hypothesis was not very, if at all, predictive of language-to-music transfer.

Interestingly, there is growing behavioral evidence on language-to-music transfer (Wong et al., 2012; Asaridou and McQueen, 2013; Bidelman et al., 2013). Lexically, tone languages (e.g., Cantonese and Mandarin) place a heavier demand on pitch than do non-tonal languages (e.g., Dutch, English, French, and Japanese) (Cutler, 2012). Relative to non-tonal language listeners, tone language listeners consistently showed superior performance on musical pitch perception tests. In the Online Identification Test of Congenital Amusia, Cantonese listeners outperformed English and French listeners on musical pitch perception (Wong et al., 2012). Even when non-verbal intelligence and working memory were controlled, Cantonese listeners outperformed English non-musicians on self-designed musical pitch memory and discrimination tasks (Bidelman et al., 2013). This further indicated that tone language experience enhanced not only basic auditory sensitivity but also complex music perception. Besides Cantonese listeners, there were similar findings from other tonal populations, e.g., Mandarin listeners. In the Montreal Battery of Evaluation of Amusia, Mandarin listeners discriminated pitch more accurately than did Dutch listeners (Chen et al., 2016). In the melody subtest of the well-validated Musical Ear Test, Mandarin listeners scored higher than Japanese listeners (Zhang et al., 2020). Collectively, the above studies have suggested that speaking a tone language sharpens musical pitch sensitivity.

Beyond behavioral advantages, language-to-music transfer also enhances the neural encoding of musical pitch (Bidelman et al., 2010, 2011). Bidelman et al. (2010) compared English musicians, English non-musicians, and Mandarin non-musicians on their FFR to musical pitch interval and Mandarin tone. Relative to the English non-musicians, the Mandarin non-musicians showed a higher pitch tracking accuracy on musical pitch interval. In line with the OPERA hypothesis, this result suggested that tone language experience enhanced the subcortical encoding of musical pitch (Patel, 2011, 2014). Could this enhanced neural encoding explain the behavioral advantage enjoyed by tone language speakers on musical pitch perception? In a later study, Bidelman et al. (2011) tested English musicians, English non-musicians, and Mandarin non-musicians on behavioral and neural perception of musical pitch. While the Mandarin non-musicians showed stronger FFR than English non-musicians, the former did not outperform the latter on behavioral musical pitch discrimination. This seemed to indicate that although tone language experience enhanced the subcortical processing of musical pitch, this neural enhancement did not yield any behavioral perceptual advantage. However, the results should be interpreted with caution given (a) the small sample size (*n* = 11 per group) and (b) the preponderance of studies showing that Cantonese/Mandarin non-musicians outperformed Dutch/English/French/Japanese non-musicians on behavioral measures of musical pitch perception (Wong et al., 2012; Asaridou and McQueen, 2013; Bidelman et al., 2013; Chen et al., 2016; Zhang et al., 2020). It remained unclear whether enhanced neural encoding of musical pitch could explain the behavioral advantage on musical pitch perception. However, this does not underscore the collective neural evidence that tone language experience enhanced the subcortical processing of musical pitch (Bidelman et al., 2010, 2011).

### Interactive Effects of Language and Musicianship on Speech and Music Perception

Although cross-domain transfer was well supported by empirical evidence, its exact nature has seldom been explored. Regarding music-to-language transfer, only few studies examined the interaction between tone language experience and musicianship (Cooper and Wang, 2012; Maggu et al., 2018b). Cooper and Wang (2012) investigated whether the combination of both tone language experience and musicianship would offer extra advantage above either experience. Specifically, they compared the Cantonese tone word learning proficiencies between Thai musicians, Thai non-musicians, English musicians, and English non-musicians. Resonating previous studies on music-to-language transfer, the English musicians had a greater learning success than the English non-musicians. However, music-to-language transfer was not observed among the Thai listeners. Intriguingly, the Thai musicians even tended to perform poorer than the Thai non-musicians. The authors attributed this non-additive effect to an internal conflict between linguistic and music perceptual mechanisms. In a related study, English musicians also outperformed English non-musicians on Thai tone word learning (Maggu et al., 2018b). Similar to the earlier finding, the Mandarin musicians tended to perform poorer than the Mandarin non-musicians. Interestingly, the study also included double tone language (i.e., Cantonese-Mandarin bilingual) groups. Compared with the Mandarin listeners, the Cantonese-Mandarin bilingual listeners did not exhibit any perceptual advantage. In other words, speaking an additional tone language did not provide any extra benefit on tone word learning. Taken together, the available studies showed that tone language experience influenced the effect of musicianship on tone word learning (Cooper and Wang, 2012; Maggu et al., 2018b).

Although language-to-music transfer was well supported by empirical evidence, its exact nature was not fully explored (Wong et al., 2012; Asaridou and McQueen, 2013; Bidelman et al., 2013; Chen et al., 2016; Zhang et al., 2020). In the context of music perception, it remains unclear whether and how language experience and musicianship interact. Most of the available studies only manipulated the language variable, and the lack of musician groups rendered them impossible to test the interaction (e.g., Wong et al., 2012; Chen et al., 2016; Zhang et al., 2020). One study attempted to manipulate both language (Cantonese and English) and musicianship variables, but the musician group only contained English musicians (Bidelman et al., 2013). In a similar vein, the lack of Cantonese musicians made it impossible to systematically test the interaction between musicianship and language experience. A recent study compared Cantonese musicians and Cantonese non-musicians on FFR to Cantonese tones and musical pitch (Maggu et al., 2018a). Regarding music-to-language transfer, the Cantonese musicians showed stronger FFR to musical pitch than the Cantonese non-musicians. The authors concluded that the combination of Cantonese language experience and musicianship offered extra perceptual advantage on musical pitch than did either experience. However, the authors also acknowledged that the lack of English musicians and non-musicians in their study rendered it impossible to fully test the music and language interaction. Also, the previous study only included a subcortical measure, i.e., FFR, which did not always correlate with behavioral musical pitch perception (Maggu et al., 2018a; Yu and Zhang, 2018). So, it remained unclear as to how such an interaction would manifest behaviorally. Limitations aside, this study provided preliminary evidence of the interaction of music and language on musical pitch perception (Maggu et al., 2018a).

Among the studies on language-to-music transfer, one study systematically manipulated both musicianship and language experience together (Ngo et al., 2016). Vietnamese and English listeners, each split into musician and non-musician groups, were assessed with the Cochran-Weiss-Shanteau index of expertise and the Montreal Battery of Evaluation of Amusia. Importantly, the interaction between language experience and musicianship was not significant. More surprisingly, the main effect of language experience was not significant too on both musical tests. While the lack of interaction might be possible, the lack of language-to-music transfer seemed unusual given substantial previous evidence (Bidelman et al., 2013; Chen et al., 2016; Wong et al., 2012; Zhang et al., 2020). Critically, Ngo et al. (2016) only recruited eight participants per group. This very small sample size might have rendered the statistical power too small to detect any effects. Also, their Vietnamese listeners grew up in the U.S. and only half of them reported having achieved native Vietnamese proficiency. Given the above limitations, the current study re-examined the interaction between language experience and musicianship with a larger (31 participants per group) and representative (native Cantonese speakers born and raised in Hong Kong; native English speakers born and raised in the United States) sample. Given the preliminary evidence that Cantonese musicians had stronger FFR to musical pitch than Cantonese non-musicians, I anticipated an interaction between language experience and musicianship on musical pitch perception (Maggu et al., 2018a). Specifically, musicianship was expected to amplify the language-to-music transfer.

### The OPERA Hypothesis and Rhythmic Perception

As mentioned previously, rhythm is another acoustic feature shared by music and speech. Musically, rhythm represents an ordered alteration of long and short notes regardless of the absolute duration of each note. Similarly, speech rhythm represents the timing of successive vowel and consonant sequences (Hayes, 1989). Speakers of different languages use rhythm differently. Based on rhythmical properties, languages are typically categorized as stress-timed (e.g., English), mora-timed (e.g., Japanese), and syllable-timed (e.g., Cantonese) (Pike, 1945; Ladefoged, 1975). In stress-timed languages, unstressed syllables are often compressed to fit in the constant interval between stressed syllables (Nespor et al., 2011). As such, successive intervals between vowels vary rigorously (i.e., high vocalic interval variability) in these languages (Ladefoged and Johnson, 2011). In syllable-timed languages, syllables have highly similar durations, rendering the vocalic interval relatively constant (i.e., low vocalic interval variability). In mora-timed languages, contrastive vowel length characterizes mora, a syllabic sub-unit which organizes speech (Otake et al., 1993). As such, stress-timed and mora-timed languages have higher vocalic interval variabilities than syllable-timed languages (Nespor and Vogel, 1986; Warner and Arai, 2001; Grabe and Low, 2002). Cross-language differences aside, rhythm (like pitch) is a common feature of music and speech. However, the OPERA hypothesis has seldom been discussed in relation to rhythmic perception (Patel, 2012).

In a follow-up paper on refining the OPERA hypothesis, Patel (2012) raised the possibility that the OPERA hypothesis might apply to rhythmic perception. For music-to-language transfer, there was behavioral and neural evidence of enhanced speech rhythm sensitivity among musicians (Marie et al., 2011; Cason et al., 2015; Magne et al., 2016; Choi, 2021). This suggested that the OPERA hypothesis also applied to rhythmic perception, at least unidirectionally (i.e., music-to-language). Similar evidence on language-to-music transfer was scarce. Two studies investigated the effect of language experience on rhythmic perception (Wong et al., 2012; Zhang et al., 2020). In the more recent study, Zhang et al. (2020) tested Mandarin and Japanese listeners with the Musical Ear Test (Wallentin et al., 2010). The Japanese listeners outperformed the Mandarin listeners on the rhythm subtest, presumably because Japanese had a higher vocalic interval variability than Mandarin. Together with prior evidence on music-to-language transfer in rhythmic perception, this finding implied that the transfer was bidirectional (Marie et al., 2011; Cason et al., 2015; Magne et al., 2016).

Being a stress-timed language, English has a higher vocalic interval variability than Cantonese (Ladefoged, 1975; Grabe and Low, 2002). Thus, it was reasonable to hypothesize that English listeners would outperform Cantonese listeners on rhythmic perception. Counterintuitively, Wong et al. (2012) reported that English listeners and Cantonese listeners performed similarly on rhythmic perception. Concerning rhythmic perception, this finding did not support language-to-music transfer at least among English vs. Cantonese listeners. Critically, methodological issues necessitate a re-examination of this preliminary conclusion. Firstly, a ceiling effect was shown on the rhythmic measure, probably because the congenital amusia screening test was too easy for typical listeners (Peretz et al., 2008). Secondly, despite the role of non-verbal intelligence in auditory perception, such measure had not been controlled (even in the study by Bidelman et al., 2013; Tang et al., 2016; Choi, 2020; Zhang et al., 2020). Going beyond these methodological limitations, the present study adopted the Musical Ear Test, the rhythmic subtest of which did not show any ceiling effect on speakers of syllable-timed languages (Wallentin et al., 2010; Zhang et al., 2020). Similar to the aforementioned research question on musical pitch, the potential interaction between musicianship and language experience on rhythmic perception was also explored.

To broaden the OPERA hypothesis, the present study examined the interactive effects of language experience and musicianship on music perception. Of particular interest was whether and how musicianship influenced the language effects on musical pitch and rhythmic perception. Based on preliminary neural evidence, I anticipated that musicianship would amplify the language effect on musical pitch perception (Maggu et al., 2018a). In other words, Cantonese musicians were expected to outperform Cantonese non-musicians, English musicians, and English non-musicians. Regarding rhythmic perception, I expected that English musicians would outperform English non-musicians, Cantonese non-musicians, and Cantonese musicians. Given the role of non-verbal intelligence in pitch perception, it was also measured and controlled as necessary (Tang et al., 2016; Choi, 2020, 2021).

## Materials and Methods

### Participants

To abide by the social distancing rules associated with COVID-19, data collection was switched from face-to-face to online. Ethical approval was obtained from the University Human Research Ethics Committee for the research project (Ref. no. A2019-2020-0036). Thus, 62 Cantonese (24 males, 38 females), and 62 English (26 males, 34 females, and 2 undisclosed) listeners were recruited via email and Prolific,^2^ respectively. Prior to data collection, all participants completed an initial online or phone screening. All Cantonese listeners reported that they (i) were living in Hong Kong, (ii) spoke Cantonese as a first language, and (iii) had normal hearing. All English listeners reported that they (iv) were living in the United States, (v) spoke English as a first language, and (vi) had normal hearing.

Based on the pre-established criteria, musicians were individuals who (a) had received 7 or more years of continuous music training and (b) could play at least one music instrument (Choi, 2020; Choi, 2021). Non-musicians were individuals who (c) had never received more than 2 years of music training, (d) had not received any music training in the past 5 years, and (e) could not play any music instrument.

Participants were tested on an online experiment platform (Gorilla Experiment Builder)^3^ (Anwyl-Irvine et al., 2020; Tsantani and Cook, 2020; Jasmin et al., 2021). They were asked to sit comfortably in a quiet environment and wear headphones. An automatic procedure ensured that the participants were using a computer but not phones or tablets. After giving written consent, the participants filled out a language and music background questionnaire (Choi et al., 2017; Choi et al., 2019; Choi, 2021). Prior to the Musical Ear Test, the participants could test and adjust the sound volume to their satisfaction (Wallentin et al., 2010). Following a written description of the task, the Musical Ear Test began. Upon completion of the Musical Ear Test, the participants completed the digital short form of the Raven’s 2 Progressive Matrices Clinical Edition (Raven et al., 2018). Between each task, the participants were given opportunities to take breaks at their own pace. To prevent prolonged idle time, an overall experimental time limit of 120 min was set for each participant.

To test whether the participants remained attentive throughout the study, five attention-check trials were embedded in the perceptual tasks. On each attention-check trial, two identical audio stimuli were presented (see Supplementary Materials). Participants then judged whether the two sounds were different. With acoustically identical stimuli, these attention-check trials could be answered easily. To be empirically stringent, only one mistake on the attention-check trials was allowed (i.e., 80% accuracy or above). As such, one Cantonese musician, one Cantonese non-musician, and one English musician were removed from the dataset.

Offline screening of the language background questionnaires showed that three English musicians and one English non-musician had learnt Cantonese or Mandarin as a second language. These participants were excluded from the dataset. Thus, the final sample consisted of 30 Cantonese musicians, 30 Cantonese non-musicians, 27 English musicians, and 30 English non-musicians.

In the final sample, all Cantonese listeners had learnt English as a second language. This is because English language education is compulsory in Hong Kong since Grade 1. Among the English listeners, only eight reported having learnt a second language. Specifically, four English musicians and four English non-musicians learnt Farsi, Hindi, Polish, Portuguese, Punjabi, Spanish, or Urdu as a second language. None of the English listeners had learnt any tone language or resided in any tone language-speaking country. The demographic, language, and music backgrounds of all participants are summarized in Tables 1–3. The very high mean accuracies on the attention trials suggested that the participants remained attentive during the experiment (*M*_*CM*_ = 97%, *SD*_*CM*_ = 7%; *M*_*CNM*_ = 97%, *SD*_*CNM*_ = 8%; *M*_*EM*_ = 94%, *SD*_*EM*_ = 9%; *M*_*ENM*_ = 96%, *SD*_*ENM*_ = 8%).

**TABLE 1:** Age, years of music training, onset age of music training, and non-verbal intelligence of the Cantonese and English musicians and non-musicians.

Group	Chronological age in years (*SD*)	Years of music training (*SD*)	Onset age of music training (*SD*)	Non-verbal intelligence (*SD*)
CM	23.8 (4.0)	10.8 (2.9)	7.8 (3.5)	17.4 (3.3)
CNM	24.9 (5.4)	0.4 (0.7)	11.5 (6.2)	16.6 (2.8)
EM	22.5 (4.7)	10.4 (3.2)	9.0 (3.5)	16.2 (3.8)
ENM	27.8 (6.3)	0.4 (0.7)	11.6 (2.9)	14.9 (5.3)

*CM, Cantonese musician; CNM, Cantonese non-musician; EM, English musician; ENM, English non-musician.*

**TABLE 2:** Language background of the bilingual Cantonese and English musicians and non-musicians.

Group	Number of bilinguals (max)	L1 frequency of use (*SD*)	L2 frequency of use (*SD*)	L1 proficiency (*SD*)	L2 proficiency (*SD*)
CM	30 (30)	5.0 (0.0)	3.3 (0.9)	4.9 (0.4)	3.3 (0.8)
CNM	30 (30)	5.0 (0.0)	2.9 (0.9)	4.9 (0.3)	3.4 (0.6)
EM	4 (27)	4.9 (0.4)	2.2 (1.2)	5.0 (0.0)	2.7 (1.0)
ENM	4 (30)	4.8 (0.7)	2.0 (0.9)	5.0 (0.0)	2.6 (1.6)

*Frequency ranges from 0 (never) to 5 (always); Proficiency ranges from 0 (non-proficient) to 5 (highly proficient).*

**TABLE 3:** Musical experience of the musicians.

Participant	Onset age (years old)	Amount of music training (years)	First instrument	Second instrument	Third instrument
CM1	5.00	15.00	Piano		
CM2	9.00	11.00	Flute		
CM3	10.00	10.00	Violin		
CM4	8.00	7.00	Tuba	Trumpet	
CM5	9.00	17.00	Oboe	Piano	
CM6	6.00	15.00	Flute	Horn	
CM7	3.00	15.00	Piano	Viola	Recorder
CM8	3.00	14.00	Piano		
CM9	5.00	14.00	Piano		
CM10	7.00	14.00	Violin		
CM11	5.00	13.00	Piano	Flute	
CM12	5.00	13.00	Piano		
CM13	5.00	13.00	Piano		
CM14	6.00	12.00	Piano		
CM15	7.00	11.00	Piano		
CM16	10.00	10.00	Violin	Piano	Others
CM17	13.00	10.00	Guitar	Bagpipe	
CM18	7.00	10.00	Piano	Clarinet	Guitar
CM19	6.00	10.00	Drums		
CM20	8.00	10.00	Piano	Drum	
CM21	7.00	9.00	Piano	Guitar	Drum
CM22	13.00	9.00	Trumpet		
CM23	20.00	9.00	Piano		
CM24	9.00	9.00	Piano		
CM25	6.00	8.00	Piano	Guitar	
CM26	8.00	8.00	Piano		
CM27	4.00	7.00	Piano	Guitar	
CM28	9.00	7.00	Clarinet		
CM29	11.00	7.00	Violin		
CM30	10.00	7.00	Piano		
EM1	11.00	17.00	Flute	Cello	
EM2	5.00	17.00	Piano	Bass	Organ
EM3	9.00	11.00	Flute	Piccolo	Others
EM4	10.00	9.00	Clarinet	Piano	Violin
EM5	6.00	7.00	Piano	Flute	Guitar
EM6	18.00	7.00	Piano		
EM7	12.00	7.00	Saxophone		
EM8	9.00	7.00	Piano		
EM9	6.00	16.00	Violin	Piano	
EM10	14.00	15.00	Piano		
EM11	5.00	14.00	Violin	Piano	
EM13	6.00	13.00	Dilruba	Harmonium	
EM14	7.00	12.00	Piano	Ukulele	
EM15	6.00	12.00	Piano		
EM16	9.00	11.00	Flute		
EM17	7.00	11.00	Piano	Clarinet	Others
EM18	11.00	11.00	Cello		
EM19	7.00	10.00	Piano	Cello	
EM21	4.00	10.00	Guitar	Piano	
EM23	8.00	10.00	Piano	Clarinet	Others
EM24	15.00	9.00	Tuba	Trombone	Others
EM25	10.00	9.00	Keyboard	Mallets	
EM26	8.00	8.00	Saxophone	Piano	
EM27	10.00	8.00	French horn	Piano	
EM28	5.00	7.00	Piano	Clarinet	
EM29	14.00	7.00	Bass		
EM30	11.00	7.00	Saxophone	Clarinet	

### Musical Ear Test

The Music Ear Test was adopted to assess musical pitch and rhythmic discrimination (Wallentin et al., 2010). The test was validated in previous studies and strongly correlated with other musical tests such as the Montreal Battery of Evaluation of Amusia (e.g., Wallentin et al., 2010; Chen et al., 2016). It has been vastly used to measure musical aptitude in Eastern and Western populations (e.g., Chen et al., 2016; Yates et al., 2019; Zhang et al., 2020).

The melody subtest contained 52 pairs of piano-played melodic phrases. They were presented audibly with an AX paradigm. On each trial, two melodic phrases with duration of one measure were played at 100 beats per minute. The participants then judged whether the melodic phrases were different. There were 26 “same” and 26 “different” trials, each carrying one point (i.e., maximum possible score = 52). All “different” trials contained a pitch violation. On half of the “different” trials, the pitch violation also caused a pitch contour change. The melody subtest began with two practice trials with feedback. No feedback was given on the experimental trials. To test the internal consistency of the items, a Cronbach’s alpha reliability coefficient was obtained from the performances of all participants across the 52 trials. The internal consistency of the melody subtest was satisfactory (Cronbach’s α = 0.76).

The rhythm subtest contained 52 pairs of rhythmical phrases generated by 4–11 wood block beats. It had the same procedure as the melody subtest. Presented in a randomized order, 31 trials contained even subdivisions of the beat whereas 21 trials contained triplets. This resulted in varying rhythmic complexities across trials. Like the melody subtest, there were 26 “same” and 26 “different” trials and the maximum possible score was 52. On each “different” trial, there was one rhythmic change (refer to Wallentin et al., 2010, p. 189; Zhang et al., 2020, p. 387 for audio and visual illustrations). Reliability analysis showed a fair internal consistency of the rhythm subtest (Cronbach’s α = 0.64).

### Raven’s Test

The digital short form of the Raven’s 2 Progressive Matrices Clinical Edition was adopted. The digital short form contained 24 randomly selected items. On each trial, a picture with a missing pattern was presented along with five possible options. The participants then chose the option which could best complete the picture. Task administration and scoring were done according to the test manual (Raven et al., 2018). The time limit was 20 min. The same reliability analysis was conducted on the 24 items. The internal consistency was satisfactory (Cronbach’s α = 0.79).

## Results

### Preliminary Analysis

To evaluate whether the four groups matched on age and non-verbal intelligence, two-way univariate analysis of variance (ANOVAs) were conducted separately on age and non-verbal intelligence with language (Cantonese and English) and musicianship (musician and non-musician) as the between-subjects factors. Regarding age, the main effect of language was not significant, *F*(1, 113) = 0.69, *p* = 0.41. However, the main effect of musicianship, *F*(1, 113) = 11.27, *p* = 0.001, η*_*p*_*^2^ = 0.09, and the interaction between language and musicianship, *F*(1, 113) = 4.56, *p* = 0.04, η*_*p*_*^2^ = 0.04, were significant. Simple effects analysis showed that the English non-musicians were older than the Cantonese non-musicians, *F*(1, 113) = 4.52, *p* = 0.04, η*_*p*_*^2^ = 0.04. The English and the Cantonese musicians matched on age, *F*(1, 113) = 0.83, *p* = 0.36.

Concerning non-verbal intelligence, the main effect of language was significant, *F*(1, 113) = 4.21, *p* = 0.04, η*_*p*_*^2^ = 0.04, but not the main effect of musicianship, *F*(1, 113) = 2.06, *p* = 0.15. Pairwise comparison showed that the Cantonese listeners outperformed the English listeners, *F*(1, 113) = 4.21, *p* = 0.04, η*_*p*_*^2^ = 0.04. The interaction between language and musicianship was not significant, *F*(1, 113) = 0.14, *p* = 0.71. Given the group differences in age and non-verbal intelligence, these two variables were controlled in the main analysis.

### Main Analysis

**FIGURE 1:**
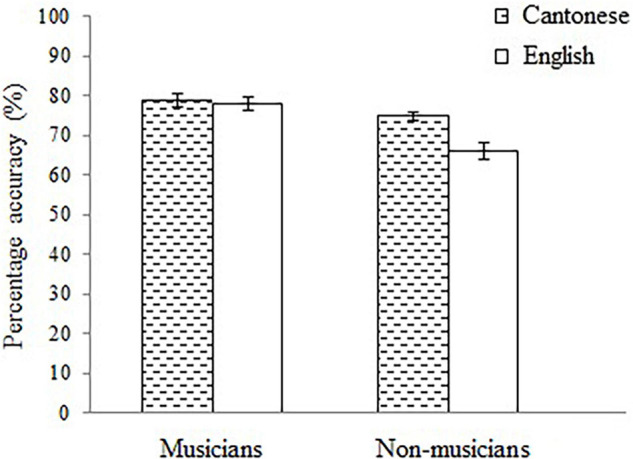
Mean pitch score of the Cantonese musicians, Cantonese non-musicians, English musicians, and English non-musicians. The error bars represent the standard error of the mean.

To ascertain whether musicianship influenced the language effect on musical pitch and rhythmic perception, a two-way MANCOVA was conducted on pitch and rhythmic scores with language (Cantonese and English) and musicianship (musician and non-musician) as the between-subjects factors, and age and non-verbal intelligence as the covariates (see Figure 1). MANCOVA revealed significant main effects of language, Λ = 0.91, *F*(2, 110) = 5.78, *p* = 0.004, η*_*p*_*^2^ = 0.10, and musicianship, Λ = 0.83, *F*(2, 110) = 11.58, *p* < 0.001, η*_*p*_*^2^ = 0.17, and the interaction between language and musicianship, Λ = 0.94, *F*(2, 110) = 3.61, *p* = 0.03, η*_*p*_*^2^ = 0.06.

Concerning musical pitch perception, there were significant main effects of language, *F*(1, 111) = 5.78, *p* = 0.02, η*_*p*_*^2^ = 0.05, and musicianship, *F*(1, 111) = 23.28, *p* < 0.001, η*_*p*_*^2^ = 0.17. Consistent with previous studies, a clear language-to-music transfer was found—knowing Cantonese seemed to offer the listeners a perceptual advantage on musical pitch perception. Expectedly, long-term musical experience also facilitated musical pitch perception. Crucially, the interaction between language and musicianship was also significant, *F*(1, 111) = 7.21, *p* = 0.01, η*_*p*_*^2^ = 0.06. This hinted that the language-to-music transfer was influenced by musicianship. Indeed, simple effects analysis revealed that the Cantonese outperformed the English listeners among the non-musicians, *F*(1, 111) = 12.97, *p* < 0.001, η*_*p*_*^2^ = 0.11, but not among the musicians, *F*(1, 111) = 0.04, *p* = 0.85. This further adds that knowing Cantonese is helpful only to non-musicians.

To further elucidate the interaction, a one-way ANCOVA was conducted on pitch score with group (Cantonese musicians, Cantonese non-musicians, English musicians, and English non-musicians) as the between-subjects factor, and age and non-verbal intelligence as the covariates. The main effect of group was significant, *F*(3, 111) = 11.21, *p* < 0.001, η*_*p*_*^2^ = 0.23. Pairwise comparisons with Bonferroni adjustments showed that the English non-musicians performed poorer than the Cantonese musicians, *p* < 0.001, Cantonese non-musicians, *p* = 0.003, and English musicians, *p* < 0.001. However, the Cantonese musicians, Cantonese non-musicians, and English musicians performed similarly, *p*s = 0.563, 0.423, 1.00.

**FIGURE 2:**
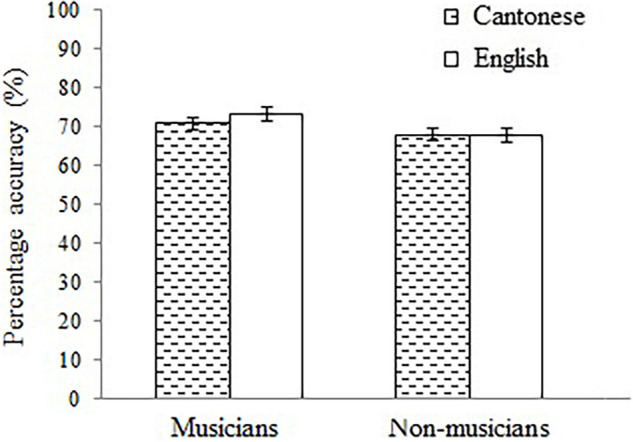
Mean rhythm score of the Cantonese musicians, Cantonese non-musicians, English musicians, and English non-musicians. The error bars represent the standard error of the mean.

Regarding rhythmic perception, the main effect of musicianship was significant, *F*(1, 111) = 6.20, *p* = 0.01, η*_*p*_*^2^ = 0.05, but not the main effect of language, *F*(1, 111) = 1.10, *p* = 0.30. The interaction between musicianship and language was not significant, *F*(1, 111) = 0.99, *p* = 0.32. Expectedly, long-term music training facilitated rhythmic perception (see Figure 2). However, language-to-music transfer was not evident in rhythmic perception, nor was any interaction.

## Discussion

The present study investigated the interactive effects of language experience and musicianship on music perception. An interaction between language experience and musicianship was found on musical pitch perception—Cantonese language experience facilitated musical pitch perception among the non-musicians but not among the musicians. Regarding rhythmic perception, the musicians consistently outperformed the non-musicians. No language or interactive effects were found.

### Musicianship Influences Language Effect on Musical Pitch Perception

The most crucial finding is that musicianship influences language-to-music transfer. It is known that tone language experience enhances musical pitch perception (Wong et al., 2012; Bidelman et al., 2013; Chen et al., 2016; Zhang et al., 2020). Strikingly, the present study found that language-to-music transfer occurred only among non-musicians. Given relevant musical experience, Cantonese language experience was no longer beneficial to musical pitch perception. This is reminiscent of two previous studies which investigated music-language interaction in an opposite direction, i.e., music-to-language transfer (Cooper and Wang, 2012; Maggu et al., 2018b). Like the present study, Cooper and Wang found significant interactive effects of musicianship and language experience (though on tone word learning). While tone language experience and musicianship each led to enhanced tone word learning, their beneficial effects did not add up. Specifically, musicians outperformed non-musicians only among the English listeners but not among the Thai/Mandarin listeners. This previous finding could be re-interpreted as such—musicianship aided tone perception only in the absence of tone language experience (Cooper and Wang, 2012; Maggu et al., 2018b). Though the present study focuses on an opposite direction, i.e., language-to-music transfer, a striking similarity was found—Cantonese language experience facilitated musical pitch perception only in the absence of long-term musical experience. Tentatively, these collective results indicate the potential need to add a new condition “**L**ack of relevant experience” to the OPERA hypothesis: For cross-domain transfer to occur, one must not possess long-term experience in the target domain.

With the potential condition “Lack” now identified, it would be interesting to see how it operates. Concerning music-to-language transfer, Thai/Mandarin musicians performed similarly as English non-musicians and even tended to perform poorer than Thai/Mandarin non-musicians (though not statistically significant; Cooper and Wang, 2012; Maggu et al., 2018b). Cooper and Wang (2012) ascribed this to an internal conflict between language and music systems. Specifically, the authors argued that music training drove Thai musicians to attend to the fine-grained acoustic details of Cantonese tones; whereas Thai language experience oriented them to ignore these details and rely on coarse tonal percepts. In the context of language-to-music transfer, the present study shows a seemingly different case. Nuanced analysis showed that the Cantonese musicians outperformed the English non-musicians. Also, the Cantonese musicians performed similarly as the Cantonese non-musicians and the English musicians. Unlike the previous studies which reflected an internal conflict, our musicians simply did not benefit from Cantonese language experience.

Speculatively, there were two possible causes for the above phenomenon. Music entails finer pitch distinction than does language (Patel, 2011, 2014). Also, musical pitch is more functionally relevant to music than to language. Conceivably, music training exerts stronger influence on musical pitch perception than does language experience. Possibly, musicianship might have already saturated the *perceptual capacity* for musical pitch or periodicity, so language experience had no effect on it. The term perceptual capacity is used here because it remains uncertain whether the saturation occurred at the cortical or subcortical levels. As mentioned above, the previous FFR study only included Cantonese musicians and non-musicians (Maggu et al., 2018a). Without measuring the FFR of English musicians, it was impossible to ascertain whether musicianship saturated the subcortical plasticity to periodicity which could have otherwise been enhanced by language experience. If the saturation occurs at the subcortical level, Cantonese musicians and English musicians are expected to show similar FFR on musical pitch perception. The other possible cause of the above phenomenon was that the musicians had developed a highly specialized cortical mechanism for musical pitch perception (Tervaniemi et al., 2006; Rogalsky et al., 2011). As such, the musicians needed not leverage on language experience for musical pitch perception. By contrast, the non-musicians might at least partially leverage on their language experience for musical pitch perception. For the Cantonese non-musicians, their linguistic experience in tone perception might have translated into perceptual benefits on musical pitch. This claim is supported by neural evidence that Cantonese non-musicians showed left hemispheric lateralization on both tone and musical pitch perception (Gu et al., 2013). To further verify this claim, future fMRI studies can examine whether musical pitch and tone perception recruit overlapping or separate cortical regions among Cantonese musicians, Cantonese non-musicians, English musicians, and English non-musicians.

At first glance, the present results contrasted the previous neural findings (Maggu et al., 2018a). On the one hand, Maggu et al. (2018a) reported that Cantonese musicians had stronger FFR to musical pitch than Cantonese non-musicians. On the other hand, our Cantonese musicians did not outperform the Cantonese non-musicians on behavioral musical pitch perception. Importantly, subcortical processing only underlies one of the many cognitive operations involved in behavioral perception (Holder, 1992; Law et al., 2013). While subcortical neural encoding is a *sine qua non*, behavioral perceptual ability may hinge on other cognitive operations. Indeed, there was evidence that FFR measures did not correlate with behavioral perception (English listeners; Yu and Zhang, 2018). Thus, enhanced FFR of Cantonese musicians does not necessarily indicate that they have a behavioral advantage on musical pitch perception.

### Bidirectional OPERA Hypothesis: Revisiting “Precision”

The present result enriches the body of evidence on language-to-music transfer (see text footnote 1) (Wong et al., 2012; Bidelman et al., 2013; Chen et al., 2016; Zhang et al., 2020). In the Musical Ear Test, the Cantonese non-musicians discriminated musical pitch height and contour more accurately than did the English non-musicians. This is consistent with previous studies showing that tone language listeners outperformed non-tonal language listeners on musical pitch perception (Wong et al., 2012; Bidelman et al., 2013; Chen et al., 2016; Zhang et al., 2020). Collectively, the present and previous findings inform the OPERA hypothesis about bidirectionality—cross-domain transfer not only occurs from music to language, but also from language to music. As mentioned in the Introduction, the OPERA hypothesis was originally devised to account for music-to-language transfer. Nevertheless, it has good potential to account for language-to-music transfer. I describe below some potential directions on how the OPERA hypothesis could be modified to broaden its coverage.

The converging evidence of language-to-music transfer, herein and in previous studies, motivates a reconsideration of how “Precision” should be defined (Wong et al., 2012; Bidelman et al., 2013; Chen et al., 2016; Zhang et al., 2020). In the original paper of the OPERA hypothesis, Precision was defined as the extent to which “a perceiver *requires* detailed information about the patterning of that feature in order for adequate communication to occur” (Patel, 2011; p. 7). In terms of the grain size, a pitch movement of only one semitone is structurally important in music (e.g., from C to C#) but not in Cantonese tones (Chow, 2012). By contrast, Cantonese tonal variations typically involve more than three semitones (Yiu, 2013). Regarding the word “*requires*”, neutralizing tonal information in Mandarin sentences did not impede comprehension among native Mandarin listeners (Patel et al., 2010). As such, Patel (2011) argued that language processing could hardly entail a high precision relative to music processing.

With its original definition of Precision, the OPERA hypothesis was not very (if at all) predictive of language-to-music transfer (Patel, 2011, 2012; Asaridou and McQueen, 2013; Patel, 2014). This was because tone perception hardly requires more precision on periodicity encoding than musical pitch perception (Patel, 2011, 2014). The present study found robust evidence that, although Cantonese tone perception required less precision than musical pitch perception, Cantonese language experience enhanced musical pitch sensitivity. Together with similar previous findings, this finding implies the need to revisit the definition of Precision. As described above, Patel (2011) viewed Precision as domain-relative, i.e., music vs. language, in which music always prevailed. Critically, the present study indicates that Precision should be re-referenced on listeners—relative to English listeners, Cantonese listeners engaged in more precise pitch perception in their first language (due to lexical tones); this precision positively transferred to the music domain. This new specification also applies potently to music-to-language transfer—musicians had more precise musical pitch perception than non-musicians; and this precision aided lexical tone perception. This new conception of Precision may help the OPERA hypothesis cover bidirectional, and more specifically, language-to-music transfer.

### Absence of Language-to-Music Transfer on Rhythmic Perception

Regarding rhythmic perception, the present study found no evidence of language-to-music transfer. Originally, it was hypothesized that the English listeners would outperform the Cantonese listeners since English had a higher vocalic interval variability than Cantonese (Nespor and Vogel, 1986; Warner and Arai, 2001; Grabe and Low, 2002). However, no significant main effect of language was shown, indicating that English language experience did not lead to better performance beyond Cantonese language experience. In fact, this finding is consistent with a previous study which reported that Cantonese and English listeners performed similarly on rhythmic perception (Wong et al., 2012). The present study has further added that task easiness does not explain the lack of group difference, because the rhythmic subtest of the Musical Ear Test showed no ceiling effect.

There are two possible explanations for the lack of language-to-music transfer in rhythmic perception. Firstly, a previous study showed that bilinguals having learnt two languages with different rhythmic properties (syllable-timed Turkish and stress-timed German) had enhanced rhythmic perception relative to those having learnt two languages with similar rhythmic properties (stress-timed German and English) (Roncaglia-Denissen et al., 2013). In the present study, a majority (86%) of the English listeners were monolinguals. However, the Cantonese listeners in the present and previous studies were all L2 English learners, meaning that they had learnt syllable-timed (i.e., Cantonese) and stress-timed (i.e., English) languages (Wong et al., 2012). It was possible that native English language experience indeed benefited the English listeners’ rhythmic perception; but then this advantage was masked by the Cantonese listeners’ enhanced rhythmic perception associated with bilingual experience. As in many Asian countries, English language instruction is compulsory in Hong Kong, so it would not be feasible to recruit Cantonese monolinguals to verify this hypothesis.

The other possible interpretation was that English language experience simply did not lead to better rhythmic perception. Although English has a high vocalic variability, duration is not the primary acoustic cue for English vocalic contrasts, e.g., tense vs. lax and full vs. reduced (Zhang et al., 2020). As such, language-to-music transfer was absent in the present and previous studies (Wong et al., 2012). Interestingly, Japanese listeners perceived rhythm more accurately than did Mandarin listeners (Zhang et al., 2020). The authors reasoned that Japanese had better durational sensitivities due to the presence of long and short vowel contrasts. These vowel contrasts are, however, absent in English.

## Future Direction and Conclusion

Aiming to provide a broad picture of how musicianship influenced the language effect on musical pitch perception, the present study viewed musicianship as a binary variable. In reality, musicians can be further categorized as amateur or professional musicians. Future studies may adopt a more fine-grained research design (2 language × 3 music groups) to see whether musicianship and language experience interact differently between amateur and professional musicians. Although non-verbal intelligence and age can be controlled statistically, future studies are encouraged to enhance stringency by recruiting matched subjects prior to experiment.

In conclusion, the present study identified an interactive effect of language experience and musicianship on musical pitch perception. Specifically, Cantonese language experience facilitated musical pitch perception only in the absence of long-term musical experience. With similar evidence that musicianship enhanced tone perception only in the absence of tone language experience (Cooper and Wang, 2012; Maggu et al., 2018b), a new condition “**L**ack of relevant experience” could be considered for the OPERA hypothesis. Apart from the interactive effect, the present study also found evidence of language-to-music transfer. Together with previous studies, this informs the OPERA hypothesis about bidirectionality (Wong et al., 2012; Bidelman et al., 2013; Chen et al., 2016; Zhang et al., 2020). As described previously, the OPERA hypothesis was not devised for language-to-music transfer. As such, it’s current conception of Precision does not readily allow language-to-music transfer. To better account for the bidirectionality, Precision could be re-referenced on listeners (rather than domains). Clearly, this study does not speak the last word on cross-domain transfer nor music and language interaction. Future studies are needed to further inform (i) how Precision could be redefined and (ii) whether the OPERA hypothesis could evolve into the O-PEARL (Overlapping, Precision, Emotion, Attention, Repetition, *Lack*) hypothesis.

## Data Availability Statement

The datasets presented in this article are not readily available because the ethical approval does not permit data sharing. Requests to access the datasets should be directed to corresponding author.

## Ethics Statement

The studies involving human participants were reviewed and approved by the Human Research Ethics Committee Education University of Hong Kong. The patients/participants provided their written informed consent to participate in this study.

## Author Contributions

WC: conceptualization, research design, data analysis, and manuscript writing and editing.

## Conflict of Interest

The author declares that the research was conducted in the absence of any commercial or financial relationships that could be construed as a potential conflict of interest.

## Publisher’s Note

All claims expressed in this article are solely those of the authors and do not necessarily represent those of their affiliated organizations, or those of the publisher, the editors and the reviewers. Any product that may be evaluated in this article, or claim that may be made by its manufacturer, is not guaranteed or endorsed by the publisher.

## Footnotes

1 In the current study, cross-domain transfer is said to occur when experience with perceptual attributes (e.g., tones) in one domain facilitates the sensitivity to perceptual attributes in a different domain (e.g., musical pitch). For example, language-to-music transfer refers to the facilitatory effect of language experience on music perception. This definition does not reject the idea that the facilitation is mediated by subcortical sensitivity to shared acoustic features (i.e., periodicity).

2
prolific.co

3
www.gorilla.sc
